# Interleukin 23: Pathogenetic Involvement and Therapeutic Target for Ulcerative Colitis

**DOI:** 10.3390/jcm14134590

**Published:** 2025-06-28

**Authors:** Laura Parisio, Giuseppe Cuccia, Anna Giudice, Federico Carrabetta, Angelo Del Gaudio, Giuseppe Privitera, Luigi Carbone, Rocco Spagnuolo, Daniela Pugliese

**Affiliations:** 1 UOS Gastroenterologia, Ospedale Isola Tiberina Gemelli Isola, 00186 Rome, Italy; laura.parisio.fw@fbf-isola.it; 2 Department of Translational Medicine and Surgery, Università Cattolica del Sacro Cuore, 00168 Rome, Italy; giuseppecuccia24@gmail.com (G.C.); delgaudioangelo@gmail.com (A.D.G.); 3 Health Sciences Department, Magna Graecia University, 88100 Catanzaro, Italy; annagiudice97@outlook.com (A.G.); fede.carrabetta@gmail.com (F.C.); 4 Department of Biomedical Sciences, Humanitas University, via Rita Levi Montalcini 4, Pieve Emanuele, 20072 Milan, Italy; gpp.privitera@icloud.com; 5 UOC Pronto Soccorso, Medicina d’Urgenza e Medicina Interna, Ospedale Isola Tiberina Gemelli Isola, 00186 Rome, Italy; luigi.carbone@fbf-isola.it; 6 IBD UNIT-CEMAD (Centro Malattie Apparato Digerente), Medicina Interna e Gastroenterologia, Fondazione Policlinico A. Gemelli IRCCS, 00168 Rome, Italy

**Keywords:** interleukin-23, Th17 lymphocytes, monoclonal antibodies, ulcerative colitis

## Abstract

Interleukin-23 (IL-23) is a key cytokine involved in the pathogenesis of various immuno-mediated inflammatory diseases. In recent years, several drugs selectively targeting IL-23 have been developed and three of them (mirikizumab, risankizumab and guselkumab) were successfully investigated in clinical trials for ulcerative colitis (UC). All of them showed a good profile for efficacy, alleviating symptoms, and inducing endoscopic and histologic improvement, with very low incidence of adverse events. Bowel urgency also emerged as a crucial outcome from patients’ perspective in the mirikizumab trials. The correct positioning of IL-23 inhibitors in the therapeutic algorithm for UC represents a new challenge for physicians, especially because it is not guided by biomarkers or predictors of effectiveness. Moreover, no comparative clinical data exist among the available IL-23 inhibitors, although molecular differences might potentially impact their effectiveness. A role for IL-23-inhibitors may also lie in combination with drugs with different mechanisms of action for complex, multi-refractory patients. This review, focusing on UC, summarizes all the clinical data available on IL-23 inhibitors and provides a perspective on the best clinical scenarios to maximize their effectiveness.

## 1. Introduction

Ulcerative colitis (UC) is a chronic systemic immuno-mediated inflammatory disease (IMID) characterized by inflammation of the colon at various extent, with rising global prevalence, particularly in Western countries and newly industrialized regions. By 2023, a prevalence of 5 million cases was estimated worldwide, with incidence rates ranging from 8.8 to 23.1 per 100,000 person-years in North America and from 0.6 to 24.3 in Europe [1,2].

Although the exact etiology and pathogenesis of UC remain unclear, a multifactorial model based on genetic predisposition together with environmental factors seems responsible for an abnormal activation of inflammatory cascades, ultimately causing disease features [2]. Typical symptoms are represented by bloody diarrhea, tenesmus, and bowel urgency, causing a significant impairment of patients’ quality of life [3].

Over the years, the recognition of underlying mechanisms and inflammatory mediators involved in the pathogenesis of UC has led to the development of target advanced therapies, mainly monoclonal antibodies, blocking single immunological targets, such as Tumor Necrosis Factor (TNF-alpha) or, more recently, Interleukin (IL)-23 [4]. However, despite therapeutic advancements, up to 7% of patients undergo colectomy, and approximately 20% are hospitalized within five years of diagnosis. Long-term complications include an elevated risk of colorectal cancer, at about 4.5% after 20 years of disease duration, and a slightly reduced life expectancy: around 80.5 years for women and 76.7 for men in North America [1]. Among the emerging therapeutic targets, IL-23 has gained considerable attention, due to its critical pathogenetic role, mainly promoting expansion and survival of T helper (Th)-17 lymphocytes–a role also undertaken in other IMID, such as psoriasis and psoriatic arthritis, for which IL-23 inhibitors offer an effective and safe therapeutic option [5].

After the approval of ustekinumab, which targets the p40 subunit shared by IL-12 and IL-23, several therapies that selectively target the p19 subunit—which is specific to IL-23—have been developed, including brazikumab, tildrakizumab, mirikizumab (MIR), risankizumab (RZB), and guselkumab (GUS), but only the last three have been successfully investigated in clinical trials for UC [6,7,8].

Despite sharing the same molecular target, they differ in structure, including the epitope surface area and the Fc binding region, both potentially influencing clinical efficacy [9].

Data from dermatological cohorts showed that molecular properties of the inhibitory epitope, binding affinity, and pharmacokinetic properties correlate with clinical efficacy in cutaneous plaque psoriasis [10].

Currently, MIR is the only anti-IL23p19 available for treating patients with UC but, in the near future, physicians will face the challenging situation of choosing among these three drugs belonging to the same class, in the absence of data on comparative efficacy [11].

The purpose of this review is to highlight key findings regarding the efficacy and safety of IL-23 inhibitors and to explore their potential future positioning in the therapeutic algorithm of UC.

## 2. Pathogenetic Role of IL-23

T lymphocytes play an essential role in the pathogenesis of inflammatory bowel disease (IBD). Briefly, T cells are abnormally activated in response to gut microbiome antigens, causing an excessive immune and inflammatory response with a significant imbalance of T lymphocytes toward the CD4+ lineage [12,13]. IL-12, produced by antigen-presenting cells, promotes the differentiation of naive T lymphocytes toward the Th1 lineage, while IL-4 leads to the differentiation of Th2 lymphocytes [14,15]. Both Th1 and Th2 lymphocytes, through the release of cytokines, such as TNF-alpha, Interferon (IFN) -gamma, and IL-5, are responsible for the acute-phase activation of the inflammatory cascade leading to tissue damage in UC [16]. Th17, a third T Helper lymphocyte population, has been shown to play a critical role in a bimodal way: exerting an inflammatory effect with the release of inflammatory cytokines, mediated by the release of IL-17F and IL-21 and, on the other hand, exerting a protective barrier with effects on membrane integrity under homeostatic conditions, mediated by the release of IL-17A and IL-22 [17]. The dual mechanism of IL-17, exerting both pro- and anti-inflammatory activities, could be responsible for the increased risk of IBD relapse observed in patients treated with IL-17 inhibitors [18].

**Figure 1:**
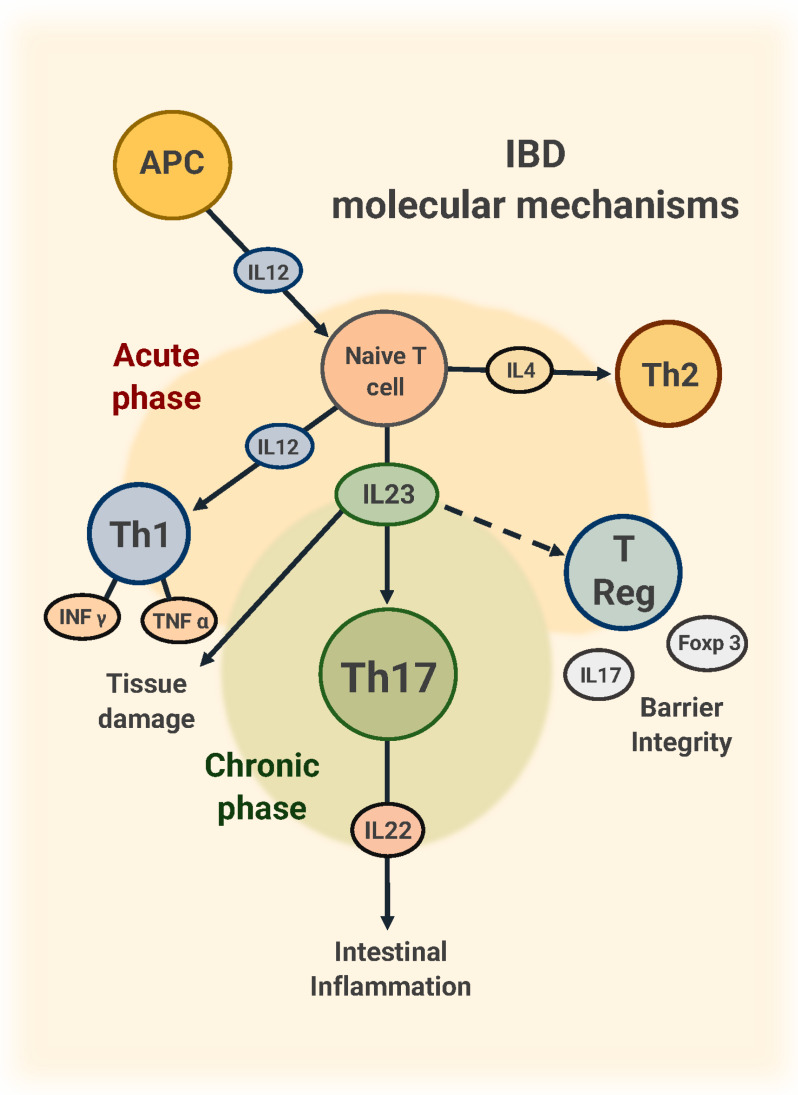
Main molecular mechanisms in IBD. Abbreviations: APC, antigen-presenting cell; IL, interleukin; Th, T helper cell.

In turn, Th17 lymphocytes’ proliferation and stabilization are maintained by the release of IL23, a pro-inflammatory heterodimeric cytokine belonging to the IL12 family and consisting of two subunits, p40 and p19 (Figure 1) [19]. The p40 subunit—also present in IL-12—engages to the IL-12Rβ1 receptor, whereas the p19 subunit specifically interacts with the IL-23R receptor. The IL23R receptor is expressed by T helper lymphocytes, innate lymphoid cells (ILC) 3, monocytes, macrophages, and dendritic cells [20,21]. The p19 subunit seems to be mainly involved in the pathogenesis of intestinal inflammation, while the p35 subunit, specific to IL-12, has a role in systemic inflammation [22].

The role of the p19 subunit seems to be more prominent in the pathogenesis of colitis compared to p35. In an animal model of colitis based on IL-10-deficient mice infected with Helicobacter hepaticus, responsible for the induction of a Th1-mediated response, mice lacking p35 expression showed severe intestinal inflammation, while selective knock-out of p19 attenuated the intensity of inflammation [23].

The IL23R variant R381Q, with a reduced receptor activity on the autocrine and paracrine mechanism of IL23, was shown to be protective against IBD [24], and the absence of the IL23R results in a reduction in the proliferation of both Th1 and Th17 lymphocytes [25]. Moreover, two meta-analyses of population-based studies evaluated the effects of specific IL23R polymorphisms on the development of colitis, observing a protective effect of rs11209026, rs7517847, and rs1088967 variants and an increased risk associated with the rs11209032 polymorphism [26,27].

High levels of IL-23 induce the activation of Th17 lymphocytes, with a possible shift toward a Th1-like phenotype capable of producing IFN-gamma, which in turn promotes increased IL-23 production, thereby amplifying and sustaining a self-perpetuating mechanism [28,29]. In addition, IL-23 is responsible for increased proliferation of gamma–delta T cells, which in turn sustain intestinal inflammation, enhancing the activity of natural killer cells, increasing their ability to attack other cells, and stimulating ILC3s to produce IL-17 and IL-22 [30]. IL-23 also exerts its activity on IL-23R-positive innate immune cells, stimulating them to releasee IL-17 and IFN gamma [30].

Finally, IL-23 acts on T-reg lymphocytes; the absence of IL-23 increases the expression of the expression of FOXP3, a transcription factor expressed by Tregs, preventing the development of colitis [31]. Cells lacking FOXP3, when transferred into mouse models lacking the p19 subunit, induce inflammatory process [31]. TGF-beta has been shown to modulate IL23R expression, reducing its concentration and consequently increasing FOXP3 expression with increased T-reg lymphocytes [32,33].

## 3. Efficacy and Safety of IL-23 Inhibitors: Randomized Controlled Trials (RCTs)

### 3.1. Mirikizumab

MIR, the first in class IL-23 inhibitor approved for UC, is a humanized IgG4-type monoclonal antibody, exclusively developed for patients affected by IBD. MIR is administered as an intravenous (IV) induction phase, 600 mg every 4 weeks (q4w) until week 8, and then 200 mg q4w subcutaneously (SC) for maintenance phase [34].

The maximum plasma concentration time (Tmax) and a steady state are reached within 8 weeks after the start of SC therapy [35].

The mean apparent clearance was 0.0229 L/hour, while the mean elimination half-life was approximately 9.3 days. Similarly to endogenous immunoglobulins, MIR is metabolized through catabolic pathways into small peptides and amino acids. The location of the injection site does not influence drug absorption [35].

The efficacy and safety of MIR were assessed in the phase 3 program LUCENT, consisting of a 12-week induction (LUCENT-1) and a 40-week maintenance phase (LUCENT-2) [6]. Patients were eligible if they were affected by moderately to severely active UC, and intolerant or exposed to glucocorticoids, conventional treatment, or biologics and JAK inhibitors. Previous exposure to anti-IL 12, anti-IL 23, and anti-IL 12/23 was an exclusion criteria.

In the LUCENT-1, patients were randomly assigned in a 3:1 ratio to receive MIR (300 mg IV induction dosing at baseline, weeks 4 and 8) or placebo. At week 12, patients achieving clinical response, underwent re-randomization and were assigned in a 2:1 ratio to receive blinded 200 mg SC MIR or placebo in the LUCENT-2 [6]. Patients not achieving clinical response at the end of induction or losing response during maintenance phase could receive an extended induction or a rescue therapy (three open-label extra infusions of 300 mg IV MIR).

The primary end points for both induction and maintenance studies were clinical remission, at week 12 and 40, respectively, both defined as a modified Mayo stool-frequency sub-score of 0, or a stool-frequency sub-score of 1 with a decrease of at least 1 point from baseline, a rectal-bleeding sub-score of 0, and an endoscopic Mayo sub-score of 0 or 1 (excluding friability) [36,37].

Secondary induction endpoints were clinical response, endoscopic remission, histologic–endoscopic mucosal improvement (HEMI), remission of symptoms, and an improvement in bowel-movement urgency. Of note, the LUCENT program was the first to evaluate urgency in UC, and urgency improvement was defined as any reduction in the Urgency Numeric Rating Scale (NRS) [38]. For maintenance study, glucocorticoid-free clinical remission, maintenance of clinical remission, endoscopic remission, histologic–endoscopic mucosal remission (HEMR) and bowel urgency remission were also assessed.

Interestingly, the LUCENT studies included treatment endpoints that combined histology and endoscopy assessments (HEMI and HEMR). Indeed, previous studies showed that improvement or resolution of inflammation measured by histological endoscopic measures together, compared with endoscopic measures alone, is strongly associated with lower rates of clinical relapse, corticosteroid use, hospitalization, and colectomy [39,40,41].

Overall, 868 patients were enrolled in the LUCENT-1 and, among them, almost 40% had been previously exposed to advanced therapies (biologic therapies and/or JAK inhibitors). Clinical remission was significantly higher in patients treated with MIR at weeks 12 and 40 (24.2% vs. 13.3%; *p* < 0.001 and 49.9% vs. 25.1%, *p* < 0.001, respectively) (Table 1). Interestingly, a total of 272 patients did not have a response to MIR therapy at the end of induction and then received open-label MIR extended induction therapy; of these, 53.7% achieved clinical response at week 24.

MIR outperformed placebo for all secondary endpoints at both timepoints (Table 2). With regard to bowel urgency, there was a significant reduction in the Urgency NRS in the MIR group compared to placebo (−2.6 vs. −1.6 at week 12; −3.8 vs. −2.7 at week 40; *p* < 0.001) at both weeks 12 and 40. The performance of MIR was significantly better than placebo, also when considering advanced-therapy-naive and -exposed patients separately.

In particular, in the naive group, at week 12 and 40, clinical remission was achieved for 30.9% and 51.5% in the MIR group vs. 15.8% and 30.7% in the placebo group, respectively; conversely, in the exposed group, rates of clinical remission recorded were 15.2% and 46.9% in of MIR group versus 8.5% and 15.6% in the placebo group, respectively.

In the LUCENT-2 trial, patients who did not achieve a clinical response at week 12 received three additional doses of MIR 300 mg every 4 weeks, for a total of 24 weeks of induction. Among these (272), 52.9% (144 patients) achieved clinical response and received MIR in the maintenance phase. After 52 weeks, 36.1% (52 patients) achieved clinical remission, and 43.1% (62 patients) achieved endoscopic remission [6].

After the end of LUCENT-2, 316 clinical remitters and responders entered the open-label, long-term extension study LUCENT-3 [42]. Of these, approximately 25% of patients had missing data at week 152 due to early discontinuation or being sporadically missing. Discontinuations or missing data were handled by Non-Responder Imputation (NRI), modified NRI (mNRI), and Observed Cases (OC). We only report in this review data from LUCENT-3 obtained using mNRI as this is a balance between NRI and OC analyses, because it counts treatment discontinuation as nonresponse but addresses sporadic missing data, avoiding bias of higher or lower remission rates using OC or NRI, respectively.

In the long-term, MIR confirmed good results for all evaluated outcomes in both maintenance responders and remitters groups. In particular, among maintenance remitters, clinical remission was maintained in 49.2% of exposed patients and in 65.8% of naive patients. Endoscopic remission was also relevant at week 152 in both remitters and responders at maintenance (61% and 72% respectively). HEMR was obtained among the maintenance remitters in 65.8% of the advanced-therapy naive and 49.2% in the exposed subgroup and, among the responders, in 52.6% of naive and 42.9% of exposed group. Furthermore, bowel urgency severity continued to reduce overtime, almost up to a 4.5 point reduction at week 152 compared to week 0 of LUCENT-1.

Finally, a specific mention should be made of a sub-analysis of the phase 2 RCT exploring the impact of MIR on gene expression in colonic tissue [43]. MIR was responsible for several transcriptomic changes, upregulating genes associated with healthy epithelial function (e.g., AQP8, ABCG2), while downregulating genes linked to inflammation and tissue damage (e.g., IL1B, MMP3, S100A8). Notably, changes in gene expression strongly correlated with clinical indices and some of the genes downregulated by MIR, such as IL1B, OSMR, and CXCL6, were previously associated with resistance to anti-TNF-alpha and JAK inhibitor therapies.

### 3.2. Risankizumab

RZB, a humanized IgG1 monoclonal antibody, has received approval for use in the treatment of psoriatic disease and Crohn’s disease, and received regulatory approval for UC in the US in June 2024 [44]. From a molecular point of view, RZB has two peculiar characteristics: the largest epitope surface area and the mutated Fc region [10]. In UC, RZB is administered IV in the induction phase, at a dosage of 1200 mg at weeks 0, 4 and 8. Then, starting from week 12, an SC dose of 360 mg or 180 mg is administered every 8 weeks (q8w) for maintenance. The choice between the two dosages can be adapted according to patients’ response at the end of induction therapy, preferring higher dosages for patients with suboptimal response.

Pharmacokinetically, RZB has a time-to-peak concentration of 3–14 days and a terminal half-life of 21–28 days. The systemic clearance and the steady-state volume of distribution were estimated at 0.31 L/day and 11.2 L/day, respectively. Although there is no specific data, RZB is not supposed to undergo metabolism via hepatic cytochrome P450 (CYP) enzymes or renal elimination.

The efficacy and safety of RZB were assessed in two multicenter, randomized controlled trials (RCTs): the INSPIRE and the COMMAND studies [8].

The INSPIRE, a 12-week induction trial, consisted of two sub-studies: a phase 2b aimed to identify the appropriate induction dose for further evaluation and the following phase 3 study. A total of 977 patients were randomized in a 2:1 ratio to receive either RZB1200 mg IV or placebo at weeks 0, 4, and 8. Eligible participants were adults aged 18–80 years with an adapted Mayo score of 5–9 and an endoscopic sub-score of 2–3, who had an inadequate response or intolerance to conventional or advanced therapies (biologics and/or JAK-inhibitors and/or ozanimod). The primary endpoint was clinical remission at week 12 (defined according to adapted Mayo score) and achieved in 20.3% of patients receiving RZB compared to 6.2% in the placebo group (adjusted difference: 14.0%; 95% CI, 10.0–18.0%; *p*  <  0.001) (Table 1). Clinical response at week 12 was observed in 64.3% of RZB-treated patients versus 35.7% with placebo (*p*  < 0.001). Endoscopic improvement occurred in 36.5% of the RZB group versus 12.1% in the placebo group (*p* < 0.001). HEMI was achieved in 24.5% with RZB versus 7.7% with placebo (*p*  < 0.001). (Table 2)

Patients who achieved clinical response at 12 weeks were re-randomized in the maintenance COMMAND study with a 1:1:1 ratio to 180 mg or 360 mg of RZB SC q8w or placebo for 52 weeks. Randomization stratification was based on the history of advanced therapies failure, last RZB induction dose, and clinical remission status (per local evaluation). A rescue therapy with open-label IV RZB was administered to patients in the event of loss of clinical response (defined as a rectal bleeding score of ≥1 point than the week 0 value or an endoscopic sub-score of 2 or 3) from week 16. Clinical remission at week 52, which was the study’s primary endpoint, occurred in 40.2% of the 180 mg group, 37.6% of the 360 mg group, and 25.1% of the placebo group (*p*  < 0.001 for both comparisons) (Table 1). Of note, among patients in clinical remission at week 52, 98.6% were not taking corticosteroids.

Major secondary endpoints included clinical response, endoscopic remission, endoscopic improvement, HEMI, maintenance of clinical remission at week 0 and at week 52, corticosteroid-free clinical remission, and maintenance of endoscopic improvement at week 0 and at week 52 (Table 2). Interestingly, endoscopic improvement was observed in 51% and 48% of RZB-treated patients (180 and 360, respectively) versus 32% in the placebo group (*p* <  0.001); HEMI occurred in 42.8% and 42.2% vs. 23.5% of placebo (*p*  <  0.001). Of note, a higher proportions of patients treated with RZB SC 180 mg were able to sustain clinical remission from week 0 to week 52 compared to placebo (70.2% vs. 39.6%, respectively *p* =  0.003); conversely, similar findings were not achieved for patients treated with the highest dose of RZB (50.0%, *p* = 0.22).

In conclusion, a higher proportion of patients treated with RZB SC 180 mg with endoscopic improvement at the beginning of COMMAND were able to maintain this up to week 52 compared to placebo-treated patients (73.6% for 180 mg vs. 47.4% for placebo, *p* = 0.002).

Among 605 patients who received RZB, 209 patients without clinical response at week 12 of induction were re-randomized to receive an additional 12 weeks of intravenous RZB 1200 mg or subcutaneous 180 mg or 360 mg. Of these, clinical remission at week 24 was achieved in 8.8% of patients with intravenous RZB and 12.7% and 15,7% with subcutaneous doses, respectively; endoscopic remission was achieved in 1.5%, 8.5% and 5.7%, resepectively. Among 100 patients (56 with 180 mg and 44 with 360 mg) who received maintenance treatment in the COMMAND study, clinical remission was achieved in 17.9% and 22.8%, respectively, while endoscopic remission in 14.3% and 18.6%, respectively, after 52 weeks of treatment [45].

For both induction and maintenance studies, a post hoc analysis evaluated patients who were naive or exposed to advanced therapies separately [46]. RZB showed a superiority over placebo for both primary endpoints: clinical remission at week 12 (advanced-therapy naive 29.7% versus placebo 8.4%, *p* < 0.0001; advanced-therapy exposed 11.4% versus placebo 4.3%, *p* = 0.0083) and at week 52 (advanced-therapy naive 50.9% or 61.7% versus 31.1%, *p* = 0.057 or *p* = 0.0033, respectively; advanced-therapy exposed 36.6% or 29.5% versus 23.2%, *p* = 0.0159 or *p* = 0.2334, respectively). Moreover, endoscopic improvement was achieved in both subgroups regardless of the advanced therapy exposition status (advanced-therapy naive 47.6% versus placebo 14.2% *p* < 0.001; advanced-therapy exposed 25.9% versus placebo 10.1%, *p* < 0.001). No direct comparison between the two groups was performed in either trial.

Another post hoc analysis of both trials was performed, exploring the correlation between exposure to RZB and serum IL-22 concentrations [46]. Patients receiving RZB throughout both the induction trial and the maintenance trial showed a significant reduction of IL-22 serum levels from baseline to week 52 (−66.3% for 180 mg and −57.0% for 360 mg, *p*  <  0.001 for both comparisons). Patients treated with RZB in the induction trial but receiving placebo in the maintenance phase still showed significant reduction of IL-22 levels through week 52 (at week 4, 51.7% and −62.1% week 52, *p* < 0.001 for both comparisons) suggesting a durable effect of RZB on the IL-23 pathway. The direct relationship between UC disease activity and this cytokine level has not yet been evaluated, but previous studies showed IL-22 to be a good predictor of efficacy for IL-23 inhibition [47,48,49].

### 3.3. Guselkumab

GUS is a fully human IgG1-lambda monoclonal antibody, approved for the treatment of plaque psoriasis and psoriatic arthritis since 2016 and currently under investigation for patients with IBD [50].

GUS showed comparable binding affinity for IL-23 and potency for inhibition of IL-23-induced STAT3 phosphorylation to RZB. However, GUS showed the strongest binding to CD64 compared to other Fc gamma receptor 1 (FcγRI or CD64), whereas RZB has a mutated Fc region, and negligible binding to any FcγR [10,51].

The binding of therapeutic antibodies to CD64 is of particular interest, as CD64+ myeloid cells producing IL-23 are increased in the inflamed colon in IBD, and this binding might potentially enhance IL-23 blockade.

Pharmacokinetically, GUS has a median time to maximum plasma concentration (Cmax) ranging from 3 to 6 days depending on the dose, and a median terminal half-life (t1/2) of 15–17 days [52].

The efficacy and safety of GUS was assessed in the QUASAR program, a comprehensive, multicenter, RCT series including: a phase 2b dose-finding induction study, two phase 3 studies (induction and maintenance), and a long-term Extension (LTE) study [7,53,54]. Patients were eligible in case of moderate to severely active UC with a history of inadequate response or intolerance to conventional therapies, biologics, or JAK inhibitors. Previous use of IL-12 or IL-23 inhibitors was an exclusion criterion. In phase 2b, two doses of IV GUS 200 mg and 400 mg were tested and compared to placebo and the first one was selected for the following phase. Accordingly, in phase 3, patients were randomized in a 3:2 ratio to receive GUS IV 200 mg or placebo q4w until week 12. An extended induction with GUS IV 200 mg or GUS SC 200 mg qw4 until week 24 was reserved for patients who received placebo and those who did not respond to GUS IV at week 12, respectively. The primary endpoint was clinical remission at week 12 (defined as a Mayo Stool Frequency of 0 or 1 with no increase from baseline, an RB sub-score of 0, and a Mayo endoscopic sub-score of 0 or 1 with no friability), that was met in 23% of patients treated with GUS compared to 8% with placebo (*p* < 0.0001) (Table 1). Among secondary endpoints, clinical response was achieved in 62% of patients treated with GUS vs. 28% with placebo (*p* < 0.0001); endoscopic improvement in 27% vs. 11% (*p* < 0.0001); endoscopic remission was in 15% vs. 5% (*p* < 0.0001) and HEMI in 24% vs. 8% (*p* < 0.0001) (Table 2).

Looking separately to patients according to their treatment history, clinical remission was recorded in 32% of GUS patients vs. 12% of placebo in the advanced-therapy-naive group, and 12% vs. 4% in the advanced-therapy-exposed group (*p* < 0.0001, *p* < 0.0052 respectively). Similar superiority was observed for all endpoints in both groups, but no direct comparisons were made between the two categories.

Clinical responders at week 12 from both phase 2 and 3 studies entered in the maintenance phase and were re-randomized in 1:1:1 ratio to receive GUS SC 100 mg q8w, 200 mg q4w, or placebo. Placebo-non responders at week 12, who received GUS IV 200 mg q4w for extra 12 weeks with benefit, were also entered in the maintenance trial. The primary endpoint was clinical remission at Week 44, significantly higher in both active groups compared to placebo (50% 200 mg q4w and 45% 100 mg q8w vs. 19%, *p* < 0.0001 for both comparisons) (Table 1).

Moreover, GUS at both dosages outperformed placebo for all secondary endpoints, including endoscopic improvement (52% 200 mg q4w and 49% 100 mg q8w versus placebo 19% *p* < 0.0001), HEMI (48% 200 mg q4w and 44% 100 mg q8w versus placebo 17%, *p* < 0.0001), maintenance of clinical remission (72% 200 mg q4w and 61% 100 mg q8w versus placebo 34%, *p* < 0.0001) (Table 2). Similar findings were also confirmed in the sub-analysis exploring separately advanced-therapy naïve and exposed patients.

At week 12, 120 non-responders received subcutaneous 200 mg GUS every week for an additional 12 weeks; of these, 55% of patients achieved clinical response at week 24 [7].

Recently, data from the ASTRO study were presented at the last European Crohn’s Colitis Organization (ECCO) Meeting [55]. This phase 3 multicenter RCT aimed to evaluate the efficacy and safety of SC GUS 400 mg as induction therapy in adults with moderately to severely active UC. Overall, 418 adult patients (both advanced-therapy-naive and exposed) were randomized to receive either SC GUS 400 mg or placebo q4w for 12 weeks. The primary endpoint was clinical remission at week 12 and was achieved by 27.6% of patients in the active arm vs. 6.5% in the placebo (*p* < 0.001). Among secondary endpoints, GUS was superior to placebo in terms of clinical response (65.6% vs. 34.5% *p* < 0.001) and endoscopic improvement (37.3% vs. 12.9% *p* < 0.001). All the results were consistent across subgroups, including both advanced-therapy-naive and exposed patients. These results were then confirmed by additional data presented at the Digestive Disease Week (DDW) 2025 from the ASTRO maintenance study, where patients were randomized to receive GUS SC 200 mg q4w or GUS SC 100 q8w [56]. ASTRO results showed the efficacy of a fully SC induction and maintenance regimen through week 24 with GUS; of note, clinically meaningful benefit was observed regardless of the exposure to advanced therapies.

At DDW, 2025 data at week 92 from QUASAR LTE were also presented [54]. Overall, 87% of participants randomized to GUS at maintenance week 0 entered the LTE, and approximately 95% of participants completed treatment through to week 92. Both GUS maintenance dose regimens sustained symptomatic, endoscopic, and histologic efficacy in patients through to week 92. In particular, clinical remitters at week 0 of maintenance trial maintained clinical remission in 86% and 78% of cases in the GUS SC 200 mg arm, and in the GUS SC 100 mg arm, respectively. Endoscopic improvement and HEMI were also sustained over time from week 44 of maintenance through week 92 in both dosage arms. Of note, clinical remission was also sustained over time regardless of advanced-therapy exposure history.

## 4. Effectiveness of IL-23 Inhibitors in Real-Life Studies

Beyond the confines of RCTs, real-life studies provide complementary insights into treatment effectiveness and safety. To date, only MIR real-world data have been reported for UC patients.

Recently, a retrospective multicenter study conducted in Japan included 52 patients with UC who first received MIR [57]. Of note, only 11.5% of patients were naive to advanced therapies and 67.3% of patients had a history of anti TNF-alpha exposure. The primary outcome was the change in the partial Mayo score (PMS) from week 0 to 12, which significantly decreased (from a median of 5, IQR 3–6, to a median of 2, IQR 0–3, *p* < 0.001). Among secondary endpoints, clinical remission rate (PMS < 2 with rectal bleeding sub-score of 0) was achieved in 44.2% of patients, CRP remission (<3.0 mg/L) in 67.3% patients and leucine-rich alpha-2-glycoprotein (LRG) in 27.3%. A subgroup analysis was performed to compare anti TNF-alpha exposed and anti TNF-alpha naive patients, but no significant differences were found in terms of clinical remission, median PMS and CRP levels. Of note, 48.1% of patients enrolled had previous exposure to ustekinumab, in contrast to the LUCENT program, which excluded this population. Therefore, a subgroup analysis was also performed comparing the ustekinumab-exposed vs. naive patients, but no significance was reached in terms of clinical remission, median PMS and CRP levels.

Similar findings come from a multicenter, prospective study from the United States of America, enrolling 22 UC patients who were started on MIR [58]. Among them, 70% of patients had been previously exposed to three or more advanced therapies. Clinical remission rate (defined as Simple Clinical Colitis Activity Index < 3 [59]) increased from 30% at baseline to 83% at week 12, while corticosteroid-free remission increased from 15% at baseline to 78% at week 12. AEs were mild to moderate, with one serious event of streptococcal pharyngitis [58].

Finally, a case series of 10 ustekinumab-exposed (1 primary non-responders and 9 secondary non-responders) patients treated with MIR was also published [60]. Corticosteroid-free clinical remission was achieved in 7 of 10 cases and, notably, 5 patients achieved clinical remission within the first month. No AEs were observed.

## 5. Safety of IL-23 Inhibitors

The majority of safety data come from RCTs, confirming the overall favourable safety profile of IL-23 inhibitors. In LUCENT-1 and 2, Serious Adverse Events (SAEs) occurred in 2–3% of patients receiving MZB [6]. Similarly, in the long-term in LUCENT-3, 7.4% of patients experienced treatment-related severe AEs, but only 8.8% SAEs and 5.3% discontinued therapy due to an AE [42]. The most common treatment-related AEs were COVID-19 infection (22.4%) and worsening of UC (15.9%). Infections occurred in 42% of patients, but only 2.4% experienced serious infections; malignancies, major adverse cardiac events (MACEs), and injection site reactions occurred in 0.3%, 0.3%, and 5.6% of patients, respectively [6]. Only one death occurred and was related to thrombotic thrombocytopenic purpura. Regarding RZB, in the INSPIRE Study, SAEs occurred in 2.3% of patients compared to 10.2% in the placebo group [8]. The most common AEs in the RZB group were COVID-19 infection (4.8%) and anemia (3.4%). A good safety profile was confirmed even in the maintenance phase, with only 5.2% of patients reporting SAEs in both treatment arms, compared to 8.2% in the placebo group [8]. The most common AEs in the active group were COVID-19 infection (10.3% with 180 mg and 13.3% with 360 mg). One death occurred in the 360 mg RZB group due to colonic cancer, apparently unrelated to the study drug. No adjudicated major adverse cardiac events or anaphylactic reactions were reported (Table 3). GUS also demonstrated a good safety profile: in the induction phase, SAEs were recorded in 3% of patients, but only 7% of AEs led to treatment discontinuation; during maintenance phase, SAEs occurred in 3% (100 mg) and 6% (200 mg) of GUS-treated patients, but only 4% (100 mg) and 3% (200 mg) of AEs led to treatment discontinuation [7]. To date, no new safety concerns emerged from the ASTRO study and QUASAR LTE [53,54,56]. The distribution of AEs recorded in each RCT is reported in Table 3.

RCTs also report data on anti-IL23 agents’ immunogenicity. Concerning immunogenicity, specifically, 23% (88/378) of MIR treated patients developed anti-drug antibodies (ADA), but only in 10 patients (2.6%) was a reduction in serum trough levels recorded Moreover, the presence of ADA was not associated with adverse events (AEs) through to 152 weeks [61]. ADA and neutralizing antibodies (NABs) were detected in up to 24% and 14% of RZB-treated patients, respectively, but in most cases at low titer. No significant correlations were found between both types of antibodies and changes in RZB concentrations, clinical response or safety [44]. Finally, with regard to GUS, the presence of ADA was recorded in 6 patients (1%) and 58 patients (12%) in the induction and maintenance studies, respectively. Overall, only 9 patients had NABs, but no correlation with infusion. or injection-site reactions emerged [7].

Regarding safety, in LUCENT-1 and 2, Serious Adverse Events (SAEs) occurred in 2–3% of patients [6]. A similarly good profile was confirmed in the long-term in LUCENT-3, with 7.4% of patients experiencing treatment-related severe AEs, but only 8.8% SAEs and 5.3% discontinuing therapy due to an AE [42].

The most common treatment-related AEs were COVID-19 infection (22.4%) and worsening of UC (15.9%). Infections occurred in 42% of patients, but only 2.4% experiencing serious infections; malignancies, major adverse cardiac events (MACE), and injection site reactions occurred in 0.3%, 0.3%, and 5.6% of patients, respectively [42]. Only one death occurred and was related to thrombotic thrombocytopenic purpura.

Real-world data are currently available only for MZB. In a Japanese retrospective study, AEs were reported in 17.3% of patients, and the most common was skin rash. No AEs led to permanent discontinuation of MIR or to death [57]. Nine AEs were reported in a recent observational study from the U.S., the most common being pain at injection site (n = 3); furthermore, one serious AE was observed, and no AE led to therapy discontinuation [58]. Finally, in a case-series including ustekinumab-exposed patients treated with MZB, no AEs were reported [60].

Overall, current data support the favourable safety profile of all anti-IL23 agents. Infections are the most common AEs, but most are mild and do not require permanent drug discontinuation; furthermore, injection site reactions have been reported for RZB and MZB, but not for GUS in RCTs. Immunogenicity does not appear to be a clinically relevant problem, as the development of ADAs seemingly does not correlate to either reduced effectiveness or increased risk of AEs. Data from LTE and real-world will be crucial to evaluate the long-term safety profile, especially in regard to rare and slow-onset AEs (such as cardiovascular accidents and cancers).

## 6. Positioning of IL-23 Inhibitors

The correct positioning of IL-23 inhibitors in the therapeutic algorithm of moderate-to-severe UC still represents a big challenge for physicians. Currently, no head-to-head studies comparing this class with other advanced therapies exist and no specific biomarkers or clinical predictors of effectiveness have been identified so far. The American Gastroenterological Association (AGA) Living Clinical Practice Guidelines propose separate recommendations for best using advanced therapies, for naive and exposed patients, based on the indirect comparative effectiveness versus placebo [62]. Overall, 38 phase 2 or 3 RCTs with advanced therapies enrolling outpatients with moderate-to-severe UC were included; of these, 5 were with IL-23 inhibitors.

In naïve patients, among IL-23 inhibitors, the use of RZB and GUS versus no treatment is strongly recommended (strong recommendation, moderate certainty of evidence), while the use of MIR is conditionally recommended (conditional recommendation, moderate certainty of evidence). Specifically, RZB and GUS demonstrated the highest efficacy as first-line strategies in naive patients, whereas MIR showed intermediate efficacy. With regard to advanced-therapy exposed patients, particularly anti TNF-alpha agents, although ustekinumab emerged as the most effective option, and IL-23 inhibitors maintained an intermediate level of efficacy. However, considering the absence of a clear superiority of one drug over the others in both settings, the authors stressed the importance of several individual patient factors (e.g., age, comorbidities, frailty, pregnancy, adherence to therapies) and preferences (e.g., route of administration, ease of access) as drivers of choice [62].

Recently, Shehab et al. performed a systematic review and network meta-analysis of 36 RCTs, involving 14,270 patients, assessing clinical remission, endoscopic improvement, endoscopic remission and histological remission in both induction and maintenance phases [63]. Overall, following upadacitinib, RZB ranked highest in inducing clinical remission (Surface Under the Cumulative Ranking, SUCRA 91.4%), while GUS ranked third for both inducing and maintaining clinical remission (SUCRA 77.6% and 78.2%, respectively). With regard to endoscopic improvement, RZB ranked second in the induction phase (SUCRA 91.4%) after upadacitinib. Moving to histological outcomes, even though the definitions of remission were different across studies, RZB ranked first (SUCRA 89.4%) for induction of remission, while GUS ranked second (SUCRA 88.3%) in both induction and maintenance phases (88.3% and 89.5%, respectively) [63].

Fudman et al. proposed a practical algorithm, positioning IL-23 inhibitors alongside ustekinumab as second-line therapies for patients who had previously failed infliximab, when safety concerns limiting the use of JAK inhibitors [64]. The advantageous safety profile of this class represents, in fact, another key driver of choice. Thus, IL-23 inhibitors may also serve as a valid first-line alternative to vedolizumab and ustekinumab in patients with contraindications to TNF-alpha antagonists or for elderly or frail patients, or with multiple comorbidities, or with a history of serious infections or malignancy [64].

The indications for other IMIDs shared by IL-23 inhibitors, in particular for GUS and RZB for psoriasis and psoriatic arthritis, makes them an attractive option in UC patients with overlapping conditions. Compared to TNF-alpha inhibitors, IL-23 blockade has demonstrated superior or comparable efficacy in these settings, with a favorable safety profile. Few data exist on the effectiveness of IL-23 inhibitors in patients with concomitant IBD and psoriatic disease [65,66]. Recently, Ribaldone et al. reported data for a mixed cohort of 17 IBD patients managed in a joint gastro-enterologic-dermatologic clinic and treated with subcutaneous IL-23 inhibitors (9 RZB, 8 GUS) at the dosage approved for psoriasis [67]. After 3 months, 12 patients (70.5%) achieved steroid-free clinical remission. Only 13 reached one year of follow-up and, among them, 7 patients (53.8%) were in steroid-free clinical remission [67].

With regard to other extraintestinal manifestations (EIMs), the majority of data on the IL-23 pathway come from ustekinumab cohorts, showing its effectiveness, even though in a small number of patients, for the treatment of erythema nodosum, pyoderma gangrenosum, aphthous stomatitis and uveitis, but no beneficial effect on axial spondylarthritis [68]. Similar findings emerged also for RZB in a phase 2 RCT, resulting in no clinically meaningful improvements over placebo in patients with active ankylosing spondylitis [69].

In addition, few reassuring safety data exist for IL-23 drugs during pregnancy. RZB has been associated with uneventful outcomes in early pregnancy exposures, and similar findings have been reported for MIR, with no major congenital anomalies observed. Accordingly, there is currently no absolute contraindication to IL-23 inhibitor use during pregnancy or lactation, since their safety profile is expected to be similar to other biological drugs. However, treatment decisions should be individualized based on disease control and patient preference [68,69,70].

Regarding comparison between the three IL-23 drugs available for UC, no head-to head studies are currently available. An indirect comparison between the RCTs could be made but should be interpreted with caution due to the different design and analyzed population in the different studies.

In conclusion, existing data on efficacy for both naïve and exposed patients, and a favorable safety profile, combined with a mechanism of action relevant across various immune-mediated conditions, support a personalized approach to the use of IL-23 inhibitors, guided by individual patient’s characteristics.

## 7. Future Perspectives

Dual target therapy (DTT) represents a valid therapeutic option for patients with IBD who have experienced inadequate responses to biologic monotherapy, as well as in those presenting with concomitant refractory EIMs [71]. In the VEGA trial, Feagan et al. evaluated the efficacy and safety of DTT with GUS and golimumab in patients with moderately to severely active UC [72]. The DTT regimen included SC golimumab 200 mg at week 0, followed by 100 mg at weeks 2, 6, and 10, along with IV GUS 200 mg at weeks 0, 4, and 8, then transitioning to SC GUS 100 mg q8w through to week 40. Comparator arms included monotherapy with golimumab (same induction doses and then 100 mg q4w through week 34) and GUS monotherapy (200 mg IV at weeks 0, 4, and 8, followed by 100 mg SCq8w for 32 weeks). A total of 214 patients were randomized: 71 to DTT, 72 to golimumab and 71 to GUS monotherapies. By week 12, 83% of patients in the DTT group had achieved a clinical response, a higher rate compared to those treated with golimumab (61%) and GUS (75%) monotherapy groups [72], reaching statistical significance only for the first comparison (*p* = 0.0032 and *p* = 0.2155, respectively).

By week 50, AEs—mainly respiratory infections, nasopharyngitis, neutropenia, and fever—were reported in 63%, 76%, and 65% of patients in the DTT, golimumab, and GUS groups, respectively. These findings suggest that DTT with GUS and golimumab offers superior short-term efficacy over monotherapy, with a comparable safety profile, and could represent a valid option for patients with a high burden of disease [72].

Such strategies may also be helpful in overcoming resistance to IL-23 inhibitors themselves, which can arise through several interconnected mechanisms. One major factor is cytokine redundancy—alternative pro-inflammatory pathways such as IL-6, IL-1β, TNF-α, and IL-12 may compensate when IL-23 is blocked, sustaining inflammation via parallel signaling routes [5]. Genetic polymorphisms in the IL-23 receptor (IL23R) or upregulation of IL-23R on mucosal T cells may also contribute by enhancing sensitivity to residual IL-23 activity or diminishing therapeutic binding [73]. Additionally, a dysbiotic gut microbiota can drive chronic inflammation through persistent activation of innate immune cells, stimulating continued IL-23 and cytokine production independent of adaptive responses [74]. Another resistance mechanism involves Th17 cells and tissue-resident memory T cells (T_RM), which, once imprinted and established, may maintain pro-inflammatory activity even in the absence of IL-23 signaling [75]. Moreover, variability in pharmacokinetics and tissue drug penetration can limit the effectiveness of IL-23 blockade at sites of deep mucosal inflammation [76].

Finally, the established efficacy and versatility of IL-23 inhibitors have encouraged the development of more practical formulations, such as orally administered molecules.

Recently, Fourie et al. presented JNJ-77242113, a competitive peptide antagonist that binds to IL-23R with picomolar affinity, effectively blocking IL-23 signaling and downstream cytokine production [77]. Preclinical studies in rats demonstrated the ability of the orally administered molecule to reduce colon tissue inflammation, ex vivo IL-23-induced IL-17A production in blood, and skin inflammation, achieving similar efficacy to parenteral anti-IL-23 antibodies. The positive outcomes observed in the Phase 1 human trial led to an accelerated Phase 2b study in psoriasis, reinforcing interest in the therapeutic potential of this oral agent and also supporting its possible future application in the management of IBD [77].

Promising advances have also been made in the development of novel oral molecules capable of simultaneously inhibiting multiple pathways of the inflammatory cascade involved in IBD pathogenesis, aligning with the therapeutic rationale of DTT.

Jairath et al. recently presented at ECCO meeting 2025 the results of a Phase 1b randomized, double-blind, placebo-controlled study evaluating SOR102, a novel orally administered bispecific domain antibody targeting both TNF-alpha and IL-23, in patients with mild to severe ulcerative colitis [78]. Overall, 22 patients (most of them naive to advanced-therapies) were randomized to receive either SOR102 at 810 mg “Bis In Die” (BID) (n = 9), 810 mg “Quaque Die” (QD) (n = 7), or placebo (n = 6) for 6 weeks and 17 patients completed the study. Treatment Emergent AEs were reported in 57%, 29%, and 50% of patients receiving SOR102 BID, SOR102 QD, or placebo, respectively. The BID dosing regimen demonstrated superior efficacy over QD, in terms of reduction of the Mayo Score (−3.8), Modified Mayo Score (−2.8), and UC-100 Score (−32.6) from baseline, alongside a 100% clinical response and symptomatic remission rate.

## 8. Conclusions

The mechanism of action of the IL-23 inhibitors is promising and innovative for the treatment of UC: three IL-23 inhibitors, MIR, RZB and GUS, demonstrated efficacy in RCT in terms of clinical and endoscopic outcomes, but also of innovative endpoints, such as bowel urgency improvement or remission and HEMI or HEMR. The efficacy was seemingly not impacted by previous exposure to advanced therapies. A good safety profile and low immunogenicity also characterize this class of drugs, with the most common AEs reported being COVID-19 infections and injection site reactions. Few real-life data exist only for MIR, but with small sample size and short duration of follow-up. Currently, no direct comparison exists among IL-23 inhibitors and other advanced therapies or within the class, and no specific clinical features or biomarkers predicting clinical effectiveness have been identified. In clinical practice, the positioning of IL-23 inhibitors is based on several factors, such as prior treatment exposure, safety concerns, disease burden, EIMs, coexisting IMIDs and practical considerations (route of administration, cost and accessibility). In this complex landscape, further additional real-life studies and pragmatic trials are required to better elucidate the optimal position of IL-23 inhibitors in the therapeutic algorithm of UC.

## Figures and Tables

**Table 1:** Primary endpoint and results of the major RCT of IL-23 inhibitors.

RCT	Phase	Type	Design	Weeks	Year of Publication	Intervention (Randomization)	N^ of Patients (a)	Primary Endpoints	Results (Drug vs. PBO; *p* < 0.001)
LUCENT-1 [6]	3	Induction	Double blind	12	2023	MIR IV 300 mg vs. PBO (3:1)	868	Clinical remission	24.2% vs. 13.3%
LUCENT-2 [6]		Maintenance		40	2023	MIRSC 200 mg SC vs. PBO (2:1)	816 (responders to LUCENT-1)		49.9% vs. 25.1%
INSPIRE (b) [8]	2b/3	Induction		12	2025	RIS IV 1200 mg vs. PBO q4w (2:1)	977		20.3 vs. 6.2%
COMMAND (b) [8]	3	Maintenance		52	2025	RIS SC: 180 mg q8w vs. 360 mg q8w vs. PBO (1:1:1)	584 (clinical responders to INSPIRE)		40.2% vs. 37.6% vs. 25.1%
QUASAR [7]	3	Induction		12	2025	GUS IV 200 mg vs. PBO q4w (3:2)	701		23% vs. 8%
		Maintenance		44	2025	GUS SC: 200 mg qw4 vs. 100 q8w vs. PBO	568 (GUS induction responders and PBO to GUS crossover week 24 responders)		50% vs. 45% vs. 19%

Abbreviations: RCT, randomized clinical trial; MIR, mirikizumab; RIS, risanlizumab; GUS, guselkumab; IV, intravenous; SC, subcutaneous; PBO, placebo; q8w, every 8 weeks; q4w, every 4 weeks; (a) Randomized. (b) The primary approach for handling missing data was non-responder imputation, while incorporating multiple imputation to handle missing data due to logistical restrictions because of the COVID-19 pandemic or geopolitical restrictions for the categorical variables.

**Table 2:** Major secondary endpoints and results of the major RCT of IL-23 inhibitors.

RCT	Phase	Type	Design	Weeks	Year of Publication	Intervention (Randomization)	N^ of Patients (a)	Secondary Endpoints (Major)	Results (Drug vs. PBO; *p* < 0.001)
LUCENT-1 [6]	3	Induction	Double blind	12	2023	MIR IV 300 mg vs. PBO (3:1)	868	Clinical response Endoscopic remission HEMI Change in bowel urgency from baseline (at week 12)	63.5% vs. 42.2% 36.3% vs. 21.1% 27.1% vs. 13.9% −2.6 vs. −1.6
LUCENT-2 [6]		Maintenance		40	2023	MIRSC 200 mg SC vs. PBO (2:1)	816 (responders to LUCENT-1)	Maintenance of clinical remission Endoscopic remission HEMR Change in bowel urgency from baseline of LUCENT-1 (at week 40)	63.6% vs. 36.9% 58.6% vs. 29.1% 43.3% vs. 21.8% −3.8 vs. −2.7
INSPIRE (b) [8]	2b/3	Induction	Double blind	12	2025	RIS IV 1200 mg vs. PBO q4w (2:1)	977	Clinical response Endoscopic remission Endoscopic improvement HEMI	51.7% vs. 18.3% 10.6% vs. 3.4% 36.5% vs. 12.1% 24.5% vs. 7.7%
COMMAND (b) [8]	3	Maintenance	Double blind	52	2025	RIS SC: 180 mg q8w vs. 360 mg q8w vs. PBO (1:1:1)	584 (clinical responders to INSPIRE)	Clinical response Endoscopic improvement Endoscopic remission HEMI Steroid-free clinical remission	68.2% vs. 62.3% vs. 51.9% 50.8% vs. 48.3% vs. 31.7% 23.2% vs. 24.3% vs. 14.8% (*p* 0.01) 42.8% vs. 42.2% vs. 23.5% 40% vs. 37% vs. 25% (*p* < 0.01)
QUASAR [7]	3	Induction	Double blind	12	2025	GUS IV 200 mg vs. PBO q4w (3:2)	701	Clinical response Endoscopic improvement Endoscopic remission HEMI	62% vs. 28% 27% vs. 11% 15% vs. 5% 24% vs. 8%
		Maintenance		44	2025	GUS SC: 200 mg qw4 vs. 100 q8w vs. PBO	568 (GUS induction responders and PBO to GUS crossover, week 24 responders)	Maintenance of clinical remission Endoscopic improvement Endoscopic remission HEMI	72% vs. 61% vs. 34% 52% vs. 49% vs. 19% 34% vs. 35% vs. 15% 48% vs. 44% vs. 17%

Abbreviations: RCT, randomized clinical trial; MIR, mirikizumab; RIS, risanlizumab; GUS, guselkumab; IV, intravenous; SC, subcutaneous; PBO, placebo; q8w, every 8 weeks; q4w, every 4 weeks; HEMR, Histologic endoscopic mucosal remission; HEMI, Histologic—endoscopic mucosal improvement. (a) Randomized. (b) The primary approach for handling missing data was non-responder imputation, while incorporating multiple imputation to handle missing data due to logistical restrictions because of the COVID-19 pandemic or geopolitical restrictions for the categorical variables.

**Table 3:** Overall summary of adverse events from the trial maintenance studies.

	LUCENT-3 Open-Label Extension Study-152 Weeks [42]	QUASAR Phase 3 Double-Blind Study-44 Weeks [7]			COMMAND Phase 3 Double-Blind Study-52 Weeks [8]		
Drug	Mirikizumab	Guselkumab			Risankizumab		
Dose	200 mg Q4W	Placebo	100 mg Q8W	200 mg Q4W	Placebo	180 mg Q8W	360 mg Q8W
Patients (N)	339	192	186	190	196	193	195
AE Total	250 (73.7%)	131 (68%)	120 (65%)	133 (70%)	150 (76.5%)	140 (72.5%)	138 (70.6%)
Serious AE (%) *	30 (8.8%)	1 (1%)	5 (3%)	12 (6%)	16 (8.1%)	10 (5.1%)	10 (5.1%)
AE leading to study drug discontinuation	18 (5.3%)	13 (7%)	7 (4%)	5 (3%)	3 (1.5%)	3 (1.5%)	5 (2.5%)
Death	1 (0.3%)	0	0	0	0	0	1 (0.5%)
COVID-19 infections	76 (22.4%)	27 (14%)	24 (13%)	18 (9%)	27 (13.7%)	20 (10.3%)	26 (13.3%)
Serious infections	8 (2.4%)	0	1 (1%)	2 (1%)	4 (2%)	2 (1%)	1 (0.5%)
Opportunistic infection	6 (1.8%)	0	0	0	0	0	1 (0.5%)
Hypersensitivity	4 (1.2%)	0	0	0	10 (5.1%)	20 (10.3%)	10 (5.1%)
Injection site reactions	19 (5.6%)	0	0	0	2 (1%)	7 (3.6%)	5 (2.5%)
Hepatic events	11 (3.2%)	0	0	0	1 (0.5%)	3 (1.5%)	13 (6.6%)
MACE	1 (0.3%)	0	0	1 (1%)	0	0	0
Malignancies and NMSC	1 (0.3%)	4 (2%)	0	1 (1%)	2 (1%)	0	2 (1%)

Abbreviations: AE, adverse events; N, number of patients; Q4W, every 4 weeks; Q8W, every 8 weeks; MACE, Major adverse cardiovascular event; NMSC, Non-melanoma skin cancer; * Serious AE: When the patient outcome is death, when the situation is life-threatening, requires inpatient hospitalization or causes prolongation of existing hospitalization, disability or permanent damage, congenital anomaly/birth defect, required intervention to prevent permanent impairment or damage (devices), other serious (important medical events) that may require medical or surgical intervention (treatment) to prevent one of the other outcomes.

## Data Availability

Not applicable.
